# Consciousness and education: contributions by Piaget, Vygotsky and Steiner

**DOI:** 10.3389/fpsyg.2024.1411415

**Published:** 2024-08-05

**Authors:** Tania Stoltz, Ulrich Weger, Marcelo da Veiga

**Affiliations:** ^1^Department of Theory and Fundamentals of Education, Federal University of Paraná, Curitiba, Brazil; ^2^Department of Psychology, Witten-Herdecke University, Witten, Germany; ^3^Integrated Curriculum Anthroposophical Psychology, Witten-Herdecke University, Witten, Germany; ^4^Department of Philosophy and Aesthetics, Alfter, Germany; ^5^Department of Educational Sciences, Alfter, Germany

**Keywords:** consciousness, education, Piaget, Vygotsky, Rudolf Steiner, awareness, development, cognitive development

## Abstract

The objective of this study is to gain an understanding of the development of consciousness and its relationship with education based on different theoretical models—namely those by Piaget, Vygotsky and Steiner. All three of them focus on different subcomponents of the educational process and there is hence a need for integrative discussion. Piaget and Vygotsky are fundamental references in the understanding of developmental and learning processes. Steiner was a pioneer in proposing a pedagogy that progresses by integrating feeling and wanting alongside thinking in the educational process. Their theories have important similarities but also differences and these will be essential for broadening the understanding of the construction of consciousness and its relationships with education.

## 1 Introduction

The theme of consciousness in Education Sciences has gained increasing prominence. This interest is expressed in research and teaching focused on culturally responsive teaching (Schirmer and Lockman, 2022; Tsevreni, 2022), argumentation between teacher and student focused on civic consciousness (Zhadan et al., 2004; Islam, 2019; Zaiets et al., 2022) and, above all, for the development of critical consciousness (Zorrilla and Tisdell, 2016; Hamamra et al., 2021; Ilten-Gee and Manchanda, 2021; Knipe, 2021; Naidu, 2021; Upadhyay et al., 2021; Gómez and Cammarota, 2022; Hernandez and Harris, 2022; Rauf and Shareef, 2022; Sarigöz, 2023; Seider et al., 2023). The critical consciousness approach, founded by Paulo Freire, aims to develop a deep knowledge of the world, its social and political contradictions, as well as acting against oppressive elements, based on the understanding of their determinants and towards greater equity, considering reflection and action in education. In turn, the civic consciousness approach highlights the close connection of each citizen to society and is based on the idea that the true interests of society are those of its citizens.

As such, the movement around studies on consciousness in Education Sciences is mainly focused on knowledge and emancipation of socio-historical aspects, which impact more on existence permeated by oppression, and less on the study of the process of development of consciousness itself. This aspect is considered just as fundamental in education as investment in critical consciousness and it is in this sense that we have developed this study, which aims to gain understanding of the development of consciousness and its relationship with education based on differing theoretical models.

The perspectives of Piaget, Vygotsky and Rudolf Steiner have been chosen as the theoretical models. Piaget and Vygotsky are among the main theorists influencing school education (Sharkins et al., 2017). Both focus on developing consciousness in education (Zender, 1973; Zhang, 2022). Rudolf Steiner, in turn, presents a different proposal for understanding and developing consciousness based on the integration of human thinking, feeling and wanting (Gidley, 2007, 2008, 2010; Stoltz et al., 2023). In order to overcome the dichotomies between the intellectual and emotion, the material and the spiritual, body and mind, and to increase understanding about how consciousness emerges, the need exists for discussions based on differing theoretical approaches in education.

## 2 Method

This literature review is a traditional or narrative review. Initially, we chose primary sources from Piaget, Vygotsky and Steiner on consciousness and education. After mapping each author’s ideas, we performed a search on the Eric, Scopus and EBSCO databases to collect articles and books that discuss the topic of consciousness and its relationship with education in the work of the authors we researched. The analysis and reflective discussion began with each author’s trajectory regarding the topic, considering all the material selected; subsequently, we aimed to establish relationships between the authors.

### 2.1 Consciousness and education in Piaget

Piaget can be considered to be one of the most influential figures in the field of Education (Zhang, 2022). For Piaget, consciousness plays an essential role in determining behavior (Zender, 1973). Through his Genetic Epistemology he presents the process of knowledge building with effect from its genesis. In this process he considers both in the essence and in the final version of his theory the intra, inter and trans passage and the processes of grasps of consciousness (Piaget and Garcia, 1983, 1987; Stoltz, 2018). The intra, inter and trans triad refers to the process of cognitive construction, starting with intra-object relationships (sensorimotor), moving on to inter-object relationships (pre-operational and concrete operational) and culminating in trans-object relationships (formal operational). Starting from the interaction between subject and object, each stage of knowledge development, which culminates in the construction of structures (sensorimotor, pre-operational, concrete operational and formal operational), involves intra, inter, trans passages and grasps of consciousness (Piaget and Garcia, 1983).

In the final version of his work, Piaget reformulated his logic based on a logic of meanings, giving it a less extensionist character, following the path of a logic that is both intentional and extensional (Piaget and Garcia, 1987). The starting point of this logic are inferences, a proto-logical or natural deduction system, whereby the meaningful implication relationship is the fundamental logical relationship. Starting with inferences means considering intuitions, purposes and representations as underlying cognitive behaviors. Meaningful implication is a relationship between two actions, statements or operations, which have a meaning in common for a person. Attributing meanings to objects, as well as to actions, consists of interpreting them and interpretation is permeated both with complexity and always with inferences. Meaningful implications are related to the process of consciousness in Piaget’s theory.

Piaget speaks of the exteriorization or physical-causal movement and the interiorization or logical-mathematical movement in the consciousness grasping process (Piaget, 1974a,b). These two parallel movements, which arise from the observables at the object’s periphery and also from the observables at the periphery of actions on objects, move towards the centre both of the object and the subject’s actions. Grasp of consciousness is, therefore, the result of the progression of the interaction between the object’s observables and the subject’s observables (Piaget, 1974b; Stoltz, 2018). All construction of knowledge in Piaget’s model is initially the result of the subject’s interaction with the physical and social environment and is expressed through grasps of consciousness via reflecting abstractions and considering meaningful implication (Piaget, 1975, 1977a,b; Piaget and Garcia, 1987). Piaget distinguishes between different types of abstraction depending on the starting point from which the subject abstracts. Abstraction through the material properties of reality or empirical abstraction enables knowledge of the properties contained in the object. In turn, reflecting (*réfléchissante*) abstraction derives from coordination of the subject’s actions carried out on the object, which are not observable as the subject processes them internally. This type of abstraction involves two moments: a reflective and a reflexive moment. Reflective implies the transition to the level of representation of content contained in the action. Reflexive, on the other hand, implies second-order reflection, thinking about what has been obtained at the previous level, thinking about thinking, leading to a form that can be used in different contents. In the Piagetian model, attention is focussed on the subject and on its constructions, whilst not disregarding social interactions and transmission, which it considers to be a *sine qua non* condition, alongside maturing and experience, these being factors coordinated by the self-regulating process of equilibration which in turn determines the possibility of a new equilibrium arising from a situation of cognitive imbalance (Piaget, 1964, 1975, 1990/1932, 2003/1936, 2009/1945; Kesselring, 1999; Stoltz, 2018). Grasp of consciousness, for Piaget, refers to cognitive functioning, is not explained as overall enlightenment and always refers to specific actions or notions. Grasp of consciousness begins with a *savoir-faire* and moves on to the understanding of what one already knew how to do; or from a practical action, that is an autonomous type of knowledge, to the understanding of that action, which involves conceptualization, and then inverting the order, whereby understanding guides the action (Piaget, 1974a,b; Stoltz, 2018). Piaget’s (1926) first studies about language and thought contributed to the formulation of his theory of consciousness (Zhang, 2022).

Criticisms of the Piagetian model are based above all on its non-discussion of culture in the development of knowledge, even though Piaget (Piaget and Inhelder, 2003) always understands social aspects in relation to cultural aspects. The Piagetian model is criticized for understanding egocentric thinking as self-centered, deficit-oriented and needing to be overcome in order to achieve operational thinking and consciousness (Zhang, 2022). In turn, Zender (1973) states that Piaget, like Vygotsky, placed emphasis on what the child presents and not on what it lacks. Some studies have focused on understanding Piaget’s concept of consciousness (Morgado, 1998; Ferrari et al., 2001; Ferreiro, 2001; Pons and Harris, 2001; Ferreira and Lautert, 2003; Moro, 2005; Ferrari, 2009; Fávero, 2011; Pons et al., 2012; Pinheiro and Becker, 2014; Stoltz et al., 2014; Dongo-Montoya, 2017; Stoltz, 2018). However, his concept of consciousness in education, based on its most recent version (Piaget, 1974a,b, 1976), is little known and explored in scientific literature. Its use can be thought of in conjunction with the proposal for developing critical consciousness in education based on Paulo Freire (Becker, 2010, 2017; Alves, 2019).

In short, “the process of constructing grasp of consciousness is explained by meaningful implication, reflecting abstraction and equilibration. The dialectics between body and mind and between causality and implication pervade the discussions on consciousness in Piaget’s work” (Stoltz, 2018, p. 01). Reflecting abstraction concerns the knowledge acquired through the coordination of the subject’s actions on the object initially and when thinking about such coordination, constituting a second-order abstraction. The concept of equilibration refers to the progressive movement of self-regulation and the search for a new balance or understanding based on a situation of cognitive imbalance, and is a vector of development. There is no consensus in the literature regarding understanding of the concept of consciousness, since over the course of his work Piaget approached it in a slightly different way (Stoltz, 2018). Nevertheless, the final version of the concept (Piaget, 1974a,b, 1976) is the result of an evolutionary process throughout his work, which includes a variety of empirical research and theoretical reflections. In this sense, it can be seen that creative activity based on what is meaningful in one’s own existence can trigger reflections that allow the development of consciousness of one’s own activity. Such awareness, following the Piagetian model, would be the result of the interaction between the subject’s observables and the observables of the creatively produced object. This process would be facilitated by the mediator who guides the subject’s attention along two parallel paths: sometimes towards the creatively produced object, sometimes towards the coordination of actions on the object within a logic of meanings (Stoltz, 2018; Stoltz et al., 2023). In the process of developing critical consciousness, this means not only knowledge of socio-historical determinants of action, but also knowledge of how the person is linked to action produced individually or collectively in a social and cultural context. Thus, the Piagetian model of consciousness-raising emphasizes the construction of the process starting from practical action to conceptualized action through the use of semiotic resources (directing thought towards the means used and the results obtained at each moment of the process) in reflecting abstraction and having as a basis a logic of meanings, in a movement of increasing equilibration, which distances it from Cartesian dualism and brings it closer to Vygotsky. It is the educator’s role to encourage the student’s actions through experiences, studies, questionings, problems and challenges that contribute to the construction of knowledge by the student (Piaget, 1998, 2000, 2003; Stoltz and Parrat-Dayan, 2007; Stoltz, 2018). Intelligence and reality are built through actions and these in turn depend on the energy that unleashes them, the affective factor (Piaget, 1954; Vonèche and Stoltz, 2007). The educator’s mediation aimed at facilitating the process of consciousness must start from questioning what has been done in practical action, seeking to achieve consciousness of the physical and mental actions in the transformation process involved in the action.

### 2.2 Consciousness and education in Vygotsky

The essential role of consciousness in human behavior permeates all of Vygotsky’s work (Zender, 1973; Van der Veer, 1984; Liu and Matthews, 2005; Toassa, 2006; Barros et al., 2009; Bozhovich, 2009; Lordelo and Tenório, 2010; Machado et al., 2011; Zavershneva, 2012, 2014; Pedrosa, 2013; Clot, 2014; González Rey, 2016; Dafermos, 2018; Davidov, 2021). However, the concept of consciousness underwent essential changes throughout his scientific career and was never finished (Zavershneva, 2014; Davidov, 2021). Vygotsky’s publication Thought and Language is the key to understanding human consciousness from his point of view (Carvalho et al., 2010).

Consciousness as a form of self-regulation and higher mental function always has its origin in a social and cultural process. In the same way as the origin of cognition occurs in a social process, for Vygotsky (1978/1935, 2002/1934; Ratner, 1995; Van Der Veer and Valsiner, 1999) this social process can be modified through the development of consciousness in the dialectic movement between individual and culture. It is in words that we find the objectification of the monistic Vygotskian view, given that the latter adds both socially shared and felt signification, in addition to meaning resulting from the subject’s activity with signification. In Vygotsky we find a type of materialist monism, given that all cognitive development is understood based on its material basis; and in the subject this understanding is based on its own brain (Van der Veer and Valsiner, 1994).

The Vygotskian theory of mediation is especially interesting in the discussions about consciousness. Based on the understanding of it as the intervention of an intermediary element in the relationship between mankind and the world, mediation expresses the intervention of culture, its instruments, signs and symbolic systems that enable the ownership and production of knowledge (Leontiev et al., 1991; Moll, 1996; Vygotsky, 2002/1934). As such, Vygotsky presents a prospective vision of learning and development, given that what the subject already knows how to do is not so much a determinant but rather how they make use of mediators to go even further, this being the same as saying how they make the most of the help or support of an expert or someone who has more knowledge (Vygotsky and Luria, 1996). It is the educator’s role to intervene in the ZPD – Zone of Proximal Development, which is located between the student’s real development zone and their potential development zone. This means intervening between what the student already knows and is able to resolve independently – their real development zone, and what they may come to know – their potential development zone, which is determined by the resolution of problems with the help of someone more capable, whether this be the teacher or another more competent subject (Vygotsky, 1978/1935; Stoltz, 2012; Stoltz et al., 2015; Dafermos, 2018). Brushlinskii (2002) criticized Vygotsky’s concept of ZPD because it leads to exacerbated pedagogical optimism, whereby any child can be taught everything provided its ZPD is respected. The gap in the Piagetian model regarding culture being taken into consideration is discussed in Vygotsky as a mediator *par excellence* of the individual’s relationships with the world.

According to Zavershneva (2014), an evolution in the understanding of the notion of consciousness can be seen in Vygotsky’s work. Starting from an idea of consciousness as a reflection of reflexes (Vygotsky, 1925/2013), in which he proposes a model of consciousness as a transmission mechanism between systems of reflexes, Vygotsky (1930/2013) moves towards the notion of consciousness as a system of secondary connections between higher mental functions, signaling the concept of mediation in the development of higher mental processes. This model gives rise to a third model (Vygotsky, 1968/2013), which understands consciousness as a dynamic semantic system, although he did not further develop this idea due to his premature death. According to Vygotsky, the level of development of conceptual thinking becomes an indicator of the level of development of consciousness. Thus, Vygotsky (1968/2013) considers semiotic analysis to be the only suitable method for studying the structure of the system and content of consciousness.

In order to understand the evolutionary process of the notion of consciousness in Vygotsky’s work, it is necessary to remember that he began with art, prior to developing his cultural-historical psychology (Dafermos, 2018; Davidov, 2021). Art, in its different manifestations, involves emotions and feelings and is understood to enable *catharsis*, involving resolution of conflicts in a playful manner (Vigotski, 1999; Connery et al., 2010; Oliveira and Stoltz, 2010; Stoltz et al., 2015). Art always expresses the social and historic development of a people, as well as the artist’s vision of the world, but it is not limited to these functions. It is for this reason that Vygotsky, in his work Educational Psychology (Vygotsky, 1997/1926), highlights the importance of working with imagination and creativity in education. Everything depends in the first place on the teacher’s emotion, which is internalized by the student. For Vygotsky, emotions have a physiological basis and, through the development of higher mental functions, can be controlled or self-regulated. It is important to highlight that art, for Vygotsky, whose view involves working with emotions, makes possible the production of individual senses which are the fruit of the subject’s negotiation with historically produced social significations.

After 1924 and with the advent of the delimitation of his cultural-historical psychology, which is of a dialectic and Marxist basis and is, therefore, materialist, Vygotsky ceases to concern himself with the question of art and creativity in itself. Later he returns to this issue at the end of his life in the text entitled: *On the problem of the psychology of the actor’s creative work*, in his unfinished theory on emotions (González Rey, 2016). According to González Rey (2016), the third moment of Vygotsky’s work can be characterized as a return to human spiritual complexity. Zavershneva and Van der Veer (2018) characterize this last period as the theory of dynamic semantic systems and the psychology of perezhivanie, which involves the semantic construction of consciousness, where the emphasis is on the study of meaning and sense as a result of operation mediated by signs.

Dafermos (2018) states that Vygotsky ended up leaving two proposals for the unit of analysis of consciousness: meaning as a unit of analysis and the concept of perezhivanie as an indivisible unit of personality and the social environment, highlighting ambiguity regarding the unit of analysis of consciousness. Cornejo (2012) points to the fact that phenomenologically experienced meaning subordinates objective meaning to subjective meaning and raises problems with the Marxist basis of Vygotsky’s work. There is a polyphony of voices in Vygotsky’s work (Van der Veer, 1984; Dafermos, 2018), also showing a romanticist Vygotsky, more interested in describing the ever dynamic conscious experience than in imposing abstractions on this experience (Cornejo, 2012, 2015). Cornejo (2015) points to the romantic roots of Vygotsky’s holism, especially in his work Thought and Language.

On the other hand, Clot (2014, p. 128) notes that in his final years Vygotsky is inclined to understand consciousness beyond thought, not as a field contemplated by the subject, but rather as a relationship between meaning given by the socio-historical environment (meaning) and meaning recreated in activity (sense). For Vygotsky, consciousness mediates, without ceasing to be mediated (Clot, 2014).

Zavershneva (2012), like Roth (2017), points out that Vygotsky goes beyond classical Marxism, indicating specific determinants of the development of consciousness. Among these determinants is the word, which plays a fundamental role in the structure of the higher mental function, highlighting Vygotsky’s program and forming its solid core. The idea of consciousness as a relationship, as a work of connection always subject to disconnection, considers the simultaneous evolution of language, abstraction and consciousness (Clot, 2014; Zavershneva, 2016). “‘En el principio fue el acto (y no el acto fue al principio), y al final surge la palabra, y eso es lo más importante’ (L.S.). Cual es lo significado de lo que hemos dicho? ‘A mi me basta con esta consciencia’, es decir, ahora me conformo con que el problema haya sido planteado.” (Vygotsky, 1968/2013, p. 130). Mediated action, communication through signs, which involves communication and generalization, becomes central in the discussion of consciousness in the final text specifically focused on this topic (Vygotsky, 1968/2013). For Zavershneva (2014), Vygotsky was successful in describing above all the cognitive sphere of consciousness, considering meaning as the result of operations mediated by signs. However, the concepts of higher emotions, will and personality, and their relationship with consciousness, involved in Vygotsky’s (1999) last project, were not completed. According to Zavershneva (2014), between 1933 and 1934, Vygotsky began the transition from meaning to the notion of perezhivanie or experience (*Erlebnis*, in German) in the investigation of the relationships between consciousness and personality. “The more personality is developed, the better the external social world is represented in the inner world of the person. Therefore, the problem of personality eventually is the knot, in which all main threads that originate in the problem of consciousness, such as the role of speech, society, environment, the problem of freedom, and the principles of the systemic and semantic nature of psyche, weave together” (Zavershneva, 2014, p. 92).

Davidov (2021) considers dramatic Vygotsky’s attempt to look for the driving force of cognitive development in the emotional sphere of humankind, rather than in the development of its activity, which highlights the inconsistency of Vygotsky’s approach in his final years in relation to the fundamental basis of mental development: on the one hand this basis is activity, while on the other hand it is affection. While for Zavershneva (2014), from the perspective of Vygotsky, consciousness exists for as long as there is an interactive theatrical performance, in which the main participant is the personality and the multiple people behind it. In this sense, Vygotsky’s vision follows a postmodern perspective of understanding consciousness based on the unity between affective and intellectual processes and could accompany the development of critical consciousness in education.

In summary, Vygotsky moves from the study of higher mental functions to the investigation of the semantic structure and development of consciousness. In this process, the focus changes from the analysis of psychological systems to the semantic analysis of consciousness and from the problem of meanings to the problem of sense, dedicating himself, in his final year of life, to the problem of emotional experience (perezhivanie) in the study of consciousness. In this process, he ends up distancing himself from his Marxist basis and moving closer to a phenomenological approach, which has been gaining ground in education.

### 2.3 Consciousness and education in Rudolf Steiner

Steiner’s proposal is mainly based on Goethe, Schiller, Schelling, Fichte and Hegel (Clement, 2016). For example, in relation to Goethe, Steiner assumes that “The Goetheanistic cosmovision differentiates itself from the materialist cosmovision by the questions it asks; neither of the cosmovisions contradict each other, rather they complement each other. Goethe’s ideas provide the basis for the former.” (Steiner, 1926, p. 77). The themes of freedom, knowledge and art permeate Steiner’s work (Clement, 2016). Despite the recognition of Goethe’s influence, recent research has pointed out the link between Steiner’s work and ideas from German idealism, which can be seen as the realization of this vision in practical life (Traub, 2011; Clement, 2016; Sparby, 2020).

Steiner’s anthropological proposal is measured by mankind itself and the process of its development. It is based on the phenomenological observation that it is necessary for human development in terms of achieving some form of freedom, which is only attained through the development of consciousness (Steiner, 1963, 2005/1904). As in Piaget and Vygotsky, consciousness is understood to be a process. In Steiner this process is seen to be unending and involves both a material and a spiritual basis. In Steiner’s view, the cognitive process includes observation and thought. For Steiner (1987), thought goes beyond the rational intellectual, it is a productive and creative activity (Stoltz and Wiehl, 2021; Traub, 2023), which involves both analytical thinking and what he describes as intuitive thinking, that will be discussed later on. Observation leads to perception, just as thought leads to concepts. Perception always represents part of reality. Concepts are the other part of reality, obtained by thinking. Reality therefore has both a material and a conceptual part, which Steiner understands as the gate to the spiritual world. “Only when the language of the outside world is in agreement with our inner self do we achieve total reality” (Steiner, 1926, p. 253). Due to our initial cognitive organization we perceive the external world as separate from us, which has occasionally been characterized by researchers as a dualistic positioning (Clement, 2016). It is, however, just one world, which is made up of ideas and concepts that permeate matter. Human beings have the task of achieving cognition of total reality. The fundamental question in Steiner is the existence of a sense for cognitive development and this in turn is related to the development of consciousness and has to be discovered by the subject itself. This is the mark of its very freedom. This sense emerges with the development of an ethical individualism (Steiner, 1987), whereby the goals of the individual are in agreement with universal goals or whereby the logic of the subject is in agreement with the external logic that surrounds it (Steiner, 2000; Sparby, 2016, 2017; Traub, 2023). This individualism is ethical only because it is in agreement with the essence of things and requires more than rational thinking but rather intuitive thinking. Indifferent observation and contemplation come prior to the process of developing knowledge (Steiner, 1979/1886). Following this, interactive observation and thought enable the development of reason in articulation with feeling and wanting. They therefore go beyond rational logic (Steiner, 1980/1892, 2022). It is important to emphasize that this rational logic is not excluded, but instead of becoming lost in the vacuum of logical abstraction, it is necessarily reencountered in the world and acts in consonance with the choice of a goal, an ideal, that is in agreement with a universal logic that sustains the universe around us (Steiner, 1987; Schiller, 1990). The encounter with a universal logic is related to the capacity of intuitive thinking. As such, the need exists to develop the ability to be intuitive, to grasp contend that are spiritual. Contemplation and meditation are understood to enable the development of intuitive thinking (Steiner, 1996b; Veiga, 2008; Stoltz and Wiehl, 2019, 2021). If the aim of education found in Piaget and Vygotsky, ultimately, is the development of consciousness, in Piaget with the development of intellectual and moral autonomy, and in Vygotsky with the development of the intellect, affection and wanting in personality, in Steiner we see an education capable of making that aim possible effectively integrating wanting and feeling with thinking. Will and love for the object, as well as cultivating thinking about one’s contribution in the world, resulting in concrete actions coherent with the sense actively given by the subject in relation to its existence, derive from the development of an ethical individualism (Steiner, 1979/1886, 1987). For Steiner, to know is to maintain a relationship with the world, this being a relationship with meaning, involving theory and practice (Welburn, 2005; Veiga, 2010; Sparby, 2016; Stoltz et al., 2023; Traub, 2023).

Steiner’s proposal for education, materialized in his Waldorf Pedagogy, consists in working on wanting, feeling and thinking in an integrated manner (Steiner, 2005; Veiga, 2006, 2010, 2015; Veiga and Stoltz, 2014; Stoltz and Weger, 2015; Schieren, 2020). Acquisition of a sense of human autonomy brought with it a distancing from the world. In this sense, connection and participation in the world are Goetheanistic elements in Waldorf education (Schieren, 2020). In the educational process, the first seven years of life are dedicated above all to the development of wanting through sensory motor activities. Between seven and fourteen years of age greater emphasis is placed on feeling through manual activities and creative and artistic productions. Emphasis on thinking is given principally after the person is fourteen years old, during adolescence. This means that concepts are worked on above all through images and in a practical manner and then worked on later through thinking (Schieren, 2010). The educational process described facilitates the development of an ethical individuality as an expression of intellectual and moral freedom in love, bringing together truth and science and resumed in the reply to the question: what is the use of all this knowledge? (Steiner, 1980/1892).

Rudolf Steiner’s theoretical model is aimed at people’s development and this is only possible through the development of their consciousness, this being a task that is imposed on today’s mankind (Steiner, 1963, 2005/1904, 2013; Stoltz et al., 2017; Sparby, 2020; Traub, 2023). In relation to Piaget and Vygotsky, Steiner advances by definitively integrating science and art in cognitive development, as two means of having access to reality and through his proposal of ethical individualism as the aim of education (Steiner, 1994, 2004; Stoltz and Weger, 2012, 2015; Stoltz et al., 2017). Ethical individualism can be developed through the synthesis between science, art and spirituality in education (Stoltz and Veiga, 2021; Stoltz and Wiehl, 2021). Each subject is unique and is the expression of the possibility of the human being (Veiga, 2010). Another essential aspect for achieving this aim in education is the prolonged work involving the body, emotions and feelings through practical activities prior to emphasis on intellectual activity (Steiner, 1996a, 2003). But even in adolescence and the primacy of the activity of thinking, this is developed through artistic activities and this alone shows itself to be a significant differential in relation to other pedagogies and enables the development of intuition integrated with thinking (Schneider, 2006; Schleder and Stoltz, 2014).

Steiner proposes a close link between knowledge and consciousness (Sparby, 2020; Traub, 2023). Art, science and spirituality are essential to the development of knowledge and consciousness. This knowledge would assimilate into itself the creative element of the Universe (Steiner, 2014), which is related to the spiritual development of human beings. Taking into account the gradual development of consciousness parallel to the development of knowledge, Steiner recognizes the following levels: material knowledge; imaginative knowledge; inspirational knowledge and intuitive knowledge (Steiner, 1987, 1996a,b, 1998a,b, 2008, 2014; Sparby, 2017, 2020; Traub, 2023). The transition from materialistic knowledge and consciousness to imaginative and subsequent consciousness is due to the development of regular attention concentration exercises (Majorek, 2007), which enable the development of new organs of perception, in addition to physical perception. These exercises, also seen as meditative (Steiner, 1996b, 1998a, 2013), enable the revelation of a new self, different from everyday life, through access to the spiritual world.

The first level of development of consciousness is acquired through material knowledge. This is understood as common or scientific sensory knowledge, which involves: the object, which promotes impressions on the senses; the image that man forms of this object; the concept, from which man arrives at a spiritual understanding of a thing or an event and “self,” which arrives at the material and spiritual unity of the object or forms the unity between image and concept. Material consciousness depends on the physical organism, especially the human brain, to manifest itself. This level of development of consciousness can also be observed by Piaget in his theory of grasp of consciousness and by Vygotsky, when describing consciousness as a higher mental function based on mediation and self-regulation. Here Steiner (1987) mentions in particular thinking as a state of exception, which is guided by observation of thinking and thinking about thinking in the sense of passing to the first level of higher knowledge, namely imaginative knowledge.

In imaginative knowledge, as described by Rudolf Steiner, there is no longer an external sensory object, leaving one with image, concept and self. At this level of knowledge and consciousness, images appear that do not originate from the material domain, but rather from the intellectual/affective domain and the spiritual domain. The common senses: touch, smell, taste, sight and hearing remain inactive here. Initially, it is necessary to acquire the ability, through concentration exercises, to form images full of meaning without sensory impressions. Steiner (1996b) proposes a series of exercises that can lead to the development of images coming from a higher plane, without falling into illusions.

At the third level of knowledge and consciousness, images disappear, leaving only concepts and self. Man lives in a purely spiritual world. Inspiration provides impressions and self elaborates the concepts. One can compare these impressions as spiritual and non-sensory sounds. For example, the sounds that accompanied the creation of Beethoven’s ninth symphony, even though he was then deaf. The world expresses, in spiritual words, it’s being to the soul and only through them does the everyday world become comprehensible.

At the final level of knowledge and consciousness, as described by Steiner, inspiration also ceases. Only the self remains in a state of resonance with the phenomena around him. This level is expressed by the faculty of no longer being outside, but rather inside things through conceptual intuition. “The life of things in the soul is *intuition*” (Steiner, 1996b, pp. 24–25). In his work The Philosophy of Freedom, Steiner (2000, p. 105) states that: “*Intuition* is the conscious experience of a purely spiritual content, which takes place in the purely noumenal sphere. Only through intuition is it possible to understand the essence of thinking.” The highest level of consciousness denotes the ability to capture free universal contents through the world of ideas and through conceptual intuition. With effect from this level, there is union between knowledge and practical action. “As soon as I recognize such contents as the basis and starting point of my action, I take action, regardless of whether the concept was already mine before, or whether I became conscious of it at the moment of my action… (Steiner, 2000, p. 109). In ordinary life, man has only one intuition: that of his own self, which can only be experienced intimately. This is how, through intuitive knowledge, we live in all things. Self-knowledge or self-awareness is an example of intuitive knowledge. However, to penetrate things in this way one needs to step outside oneself and look at others based on them. Meditation, attention concentration exercises, contemplation of natural phenomena such as growth and withering, looking back at daily actions, feelings and thoughts in interactions with the world, as well as other paths described by Steiner (1996a), can cause psychic life to temporarily move away from the sensory world and become capable of inner work towards higher levels of consciousness. In this case and according to Steiner (1996b, 2016), moral evolution is absolutely necessary so that man does not become a hostage to elementary forces when developing his consciousness towards higher regions. To this end, he points out: 1. the conscious distinction between ephemeral and eternal, based on the idea that no one knows that which is eternal in things if they do not have deep knowledge of the ephemeral; 2. attach oneself emotionally to things that have value and usefulness and giving them more appreciation than ephemeral and insignificant things; the value of the isolated object must always be considered in its connection with a totality; develop the following abilities in psychic life: control of thoughts, control of actions, equanimity, impartiality, trust in the world around us and inner balance. The transition to higher levels of consciousness requires effort, patience and regularity in carrying out exercises, in addition to developing a moral attitude towards the world and towards oneself. Development of consciousness involves self-knowledge and self-education in a direction that satisfies the human being. Inner calm and an attitude of veneration towards knowledge superior to our own being are prerequisites for going beyond material consciousness and for the development of new, non-physical senses that promote openness to the world of concepts and ideas.

Achieving higher levels of development of consciousness is justified by the gaining of new impulses for society through intuition and which enable, based on their representation, the improvement of the fields of health, education, art, agriculture, architecture, social organizations as seen in different anthroposophical initiatives around the world (Clement, 2016). Academic research into Rudolf Steiner’s thought is relatively recent and, despite the development of criticism related to the debatable quality of Steiner’s philosophical texts, questions regarding the originality of his ideas and intentions with his work (Traub, 2011; Zander, 2011), has identified Rudolf Steiner as a thinker who combines knowledge and practical achievement with great social impact. Even though contradictions and ambivalences are observed in his work (Wagemann, 2012), it represents a movement beyond the spiritual void of modern science, an investment in aesthetic thinking (Welsch, 2003; Clement, 2016), which allows the development of a “porous self” (Taylor, 2018), capable of opening up to new possibilities of knowledge and consciousness, beyond materialist consciousness (Sparby, 2020; Traub, 2023).

In summary, Rudolf Steiner presents a multifaceted and in-depth overview regarding the development of consciousness based on self-knowledge and self-education. The possibility of consciousness of self presents itself as an indispensable condition for continuing the process of developing material consciousness towards higher levels. For Steiner, all that is being is ultimately consciousness, and consciousness is the ability to access reality that is ultimately spiritual in its origin and material in its realization. Consciousness thus defines, for Steiner, the very level of evolution of the person and humanity. Waldorf Education, founded by Rudolf Steiner, seeks to promote integrated thinking, feeling and wanting through work with art, science and spirituality, preparing people for living in the world and for conscious involvement in its transformation. This proposal for education for consciousness finds support both in studies that aim to develop critical consciousness and civic consciousness, and also in studies on Consciousness-Centred Education (Beauregard, 2020), and Consciousness-Based Education (Lagrosen and Lagrosen, 2020; Fergusson et al., 2022).

## 3 Final remarks

The objective of this study is to gain understanding of the development of consciousness and its relationship with education based on differing theoretical models: Piaget, Vygotsky and Rudolf Steiner. It can be seen that the theoretical models are in agreement in understanding consciousness as a complex and multifaceted process, bringing inspiring ideas to educational practices. On the other hand, they present different perspectives, highlighting either the intellectual cognitive aspect or the integral aspect. Piaget understands consciousness as reflective abstraction based on the movement between causality and implication within a logic of meanings; Vygotsky understands consciousness as a dynamic semantic system related to emotional experience and the social situation of development; while Steiner understands consciousness as the ability to access reality through observation, rationality and intuition. The three theorists note the need for reflection and self-reflection for the development of consciousness and link it to conceptual thinking. The research of both Piaget and Vygotsky has a materialist basis and highlights intellectual, social, affective and moral integration in the development of consciousness, which is expressed in practice. Rudolf Steiner presents research with a materialistic and spiritual basis through access to the world of ideas or the spiritual world, accessible to anyone through regular exercises of concentration of attention and meditation. This opens up a new field of research, which is broad and challenging, aimed at the development of the person and humanity through the development of their consciousness.

A more profound notion of development is illustrated through the discussion in this paper. As Valsiner pointed out, developmental changes involve novelty and irreversibility time (Valsiner, 2000). The three scholars stress a notion of development that encompasses these two main qualities of development. In the same vein, the romantic roots of Steiner and Vygotskian work are connected with Goethe’s work about the metamorphosis of plants. In this work, an interesting theoretical proposal about the notion of change and conservation of the whole is carefully described (Cornejo, 2015). Romanticism laid the basis for understanding core phenomena like human development, guided in a holistic sense. This understanding is followed by Steiner and by Vygotsky.

The three scholars described in the paper put emphasis, in different ways, on the active role of the person inside their social context, each of them with slightly different notions and points of emphasis, more individual-object-oriented, social-context-oriented, or aesthetics-oriented. The activity performed by every person constitutes a central aspect of their development. The concept of self-education in Steiner goes in this direction and some authors propose that consciousness in Vygotsky always possesses a personal and subjective dimension (Valsiner and Van der Veer, 2014). In Piaget, the child also plays an active role in his or her environment.

In view of the challenges of the current world, there is a need to expand the vision of science beyond materialistic science and to consider different theoretical-practical models in order to gain a better approach to the phenomenon of consciousness, as well as the possibility of developing it through introspection or through the first person, contributing to a more human and sensitive education. In this sense, research focused on different methods of developing consciousness in teacher training becomes fundamental and is a challenge for future research.

## Author contributions

TS: Conceptualization, Data curation, Formal analysis, Funding acquisition, Investigation, Methodology, Project administration, Resources, Validation, Visualization, Writing – original draft, Writing – review & editing. UW: Conceptualization, Data curation, Formal analysis, Investigation, Methodology, Project administration, Resources, Supervision, Validation, Visualization, Writing – review & editing, Funding acquisition. MDV: Funding acquisition, Conceptualization, Data curation, Formal analysis, Investigation, Methodology, Project administration, Resources, Supervision, Validation, Visualization, Writing – review & editing.
